# Orchid conservation: how can we meet the challenges in the twenty-first century?

**DOI:** 10.1186/s40529-018-0232-z

**Published:** 2018-06-05

**Authors:** Michael F. Fay

**Affiliations:** 1 Royal Botanic Gardens, Kew, Richmond, Surrey TW9 3AB UK; 2 School of Biological Sciences, University of Western Australia, Crawley, WA 6009 Australia

**Keywords:** Conservation priorities, Systematics, Phylogenetics, Population genetics, In situ conservation, Ex situ conservation, Integrated conservation, Mycorrhizas, Pollination, Illegal trade, CITES, Red List

## Abstract

With c. 28,000 species, orchids are one of the largest families of flowering plants, and they are also one of the most threatened, in part due to their complex life history strategies. Threats include habitat destruction and climate change, but many orchids are also threatened by unsustainable (often illegal and/or undocumented) harvest for horticulture, food or medicine. The level of these threats now outstrips our abilities to combat them at a species-by-species basis for all species in such a large group as Orchidaceae; if we are to be successful in conserving orchids for the future, we will need to develop approaches that allow us to address the threats on a broader scale to complement focused approaches for the species that are identified as being at the highest risk.

## Background

We live in exciting but challenging times for the study of biodiversity and its conservation. Novel technologies provide us with the opportunity to study species and their interactions in greater detail than ever before, but challenges associated with global change, habitat destruction, changing land use and unsustainable utilization of biodiversity make its conservation ever-more urgent, and the capacity to develop solutions at the species-level for more than a small proportion of species (e.g. those identified as being at the greatest risk) is likely be outstripped by the sheer scale and pace of change (Gale et al. 2018). In response to these challenges, scientists and conservation practitioners must make difficult choices in prioritizing their work. With c. 28,000 species, orchids are probably the second largest family of flowering plants after Asteraceae (Chase et al. 2003, 2015; Willis 2017). By the end of 2017, the IUCN Global Red List included assessments for 948 orchid species, of which 56.5% are threatened (IUCN 2017), but this leaves c. 27,000 species to be assessed for the Global Red List. Due to their complex biology, notably their interactions with mycorrhizal fungi, pollinators and host trees, orchids present particular challenges for conservation, and this is compounded by non-sustainable and often illegal collection for horticulture, medicine and food and by climate change (Fay 2015a; Gale et al. 2018).

*The need for orchid conservation is paramount if we are to leave to future generations the rich and wildly fascinating orchid legacy we all enjoy today. Without effective conservation actions…, threatening process will continue to militate against the survival of rare orchids, resulting in their continued degradation and inevitable extinction.*

In this quote from their recent book, Swarts and Dixon (2017, p. 4) encapsulated the need for urgent and effective conservation action for orchids, a group that biologists including Linnaeus and Darwin have found fascinating due to their extreme specializations (Fay and Chase 2009). In this short review, I discuss the conservation status of orchids, the threats to their continued survival and approaches to addressing the challenges relating to orchid conservation in the twenty-first century.

## Conservation status of orchids—what do we know?

With c. 28,000 species divided into five subfamilies, Orchidaceae are one of the largest and most widespread families of flowering plants, and they account for c. 8% of angiosperm species diversity (Chase et al. 2003, 2015; Willis 2017). However, only c. 1000 species have been assessed for the IUCN Global Red List to date (IUCN 2017), and an alarming 56.5% of those that have been assessed fall into one of the categories of threat (critically endangered, endangered and vulnerable). Major threats include habitat destruction and unsustainable (often illegal) harvesting, and because of their complex life histories orchids are thought to be particularly vulnerable to the effects of global environmental change (Fay and Chase 2009; Swarts and Dixon 2009a; Gale et al. 2018). All species of subfamily Cypripedioideae (the slipper orchids) were assessed for the Global Red List in a recent project, and due to a combination of habitat degradation and, in some cases, ruthless harvesting, c. 90% of species were assessed as threatened (Fay and Rankou 2016). Slipper orchids were chosen for that project because of their high profile and the expected high level of threat, but even the family-wide figure of 56.5% shows that many orchid species are threatened with extinction. Their conservation should be regarded as urgent if these iconic plants are not to decline further.

## What are the threats?

Globally, habitats and the species that occur in them are under increasing pressure. Brooks et al. (2002), for example, reported that nearly 50% of vascular plant species are endemic to 25 “hotspots” of biodiversity, each with at least 1500 endemic plant species, but all of these hotspots had lost more than two-thirds of their pristine habitat. These authors predicted that, as a result of this habitat loss, many of the endemics in these hotspots are likely to become extinct or to be threatened with extinction in the near future. In a study of a Mediterranean island, Vogt-Schilb et al. (2016) presented evidence of high turnover in species composition of orchids in communities as result of change in land use. There is also increasing evidence that global change may also be influencing species distributions (e.g. Fay 2015b), and benefits of and problems associated with assisted migration and translocations to climatically suitable localities are increasingly being discussed in relation to orchids (e.g. Ramsay and Dixon 2003; Swarts and Dixon 2009a) and more generally (Pearman and Walker 2004; Ricciardi and Simberloff 2009). Like all plants, orchid species are affected by these pressures, but due to their often complex interactions with pollinators, mycorrhizal fungi and host trees, they are likely to be at greater risk as they are dependent on other organisms that are also being affected by habitat or climatic change. Thus, orchids present greater challenges than many other plant groups.

An additional area of threat relating to orchids is unsustainable harvesting, and the full impact of this is only slowly being understood. Indiscriminate collection for horticultural collections has been documented as having a major impact on some orchids, notably species of *Cattleya*, *Laelia*, *Renanthera* and some slipper orchids (*Cypripedium*, *Paphiopedilum*, *Phragmipedium*), and in some cases these have been systematically stripped from the wild to the point of (near) extinction. However, many orchids are not collected for horticulture or are collected in such small numbers that there is unlikely to be much impact (e.g. Cribb et al. 2003; Fay 2015a).

Largely as an attempt to control illegal smuggling of these desirable orchids and because of perceived problems with identification, all orchids were placed on the appendices of the Convention on International Trade in Endangered Species (CITES) (e.g. Cribb et al. 2003), and orchids account for > 70% of the species listed on CITES. However, it is becoming increasingly clear that many orchid species are still being collected and transported across international borders, for use as medicine or food in addition to the horticultural trade, without the permits required under CITES (e.g. Fay 2015a; Hinsley et al. 2018). The extent of the illegal trade is difficult to assess, but attempts are being made to estimate the extent of non-compliance with CITES regulations (e.g. Ghorbani et al. 2014; Hinsley et al. 2017). Notable examples of poorly documented trade relate to orchids collected for traditional medicine in East Asia and for production of the foodstuffs salep in the eastern Mediterranean and the Middle East (e.g. Kreziou et al. 2016; de Boer et al. 2017) and chikanda in south-eastern Africa (e.g. Veldman et al. 2014); the development of novel DNA-based techniques is now providing the opportunity to identify the orchid species in these processed foodstuffs, and this will mean that documentation and policing of the trade will become increasingly feasible (e.g. de Boer et al. 2017). In addition to not halting illegal trade, an unintended consequence of the listing of all species of orchid on CITES has been a reduction in the collection of orchids for scientific purposes, including conservation research; (Roberts and Solow 2008).

## Conserving orchids

Orchid conservation has been the subject of many reviews; to access the older literature, the reader is pointed to reviews written by Koopowitz (2001), Cribb et al. (2003), Koopowitz et al. (2003), Dixon and Phillips (2007) and Swarts and Dixon (2009b) and many of the chapters in the volume resulting from the First International Orchid Conservation Congress held in Western Australia in 2001 (Dixon et al. 2003). Most recently, Swarts and Dixon (2017) published a book focusing on conservation techniques for terrestrial orchids. Together, these publications represent a rich resource for conservation and should be consulted by anyone with an interest in orchid conservation. In their review, Swarts and Dixon (2009b) focused on the role of botanic gardens in supporting orchid conservation scientifically and horticulturally, and Pedersen et al. (2018) stressed the close link between collection-based research and conservation.

Documentation of orchid species (how many species should be recognized, where they occur and how they are related to each other) is improving, and this information provides the necessary background for establish conservation priorities, but effective conservation will also depend on the maintenance of the essential links with animals, fungi and other plants that allow them to survive. In recognition of the importance of these interactions, “Making the links” was chosen as the theme for the 5th International Orchid Conservation Congress, held on La Réunion in 2013 (Fay et al. 2015; Fay 2016; and references therein). Six of the papers from the 6th International Orchid Conservation Congress, held in Hong Kong in 2016, also focus on these links; see Gale et al. (2018) and references therein). With orchids, perhaps more than most other plant groups, integrated conservation involving in situ and ex situ approaches and studies of pollination, mycorrhizal associations and genetics (as summarized by Swarts and Dixon (2009a) for terrestrial orchids in Western Australia) will be necessary if we are to succeed.

Species-by-species approaches to conservation will continue to be the ideal for species identified as being the highest priorities or at the greatest risk, but for a group as large as Orchidaceae, in which so little is known about population genetics, etc. for many species, we will need to complement these with other approaches that allow us to conserve groups of species that have similar threats, that are closely related or that occur sympatrically. Approaches addressing conservation of process (rather than individual species) may be appropriate in groups which are undergoing relatively evolution due to hybridization and/or polyploidization (e.g. Ennos et al. 2012). Formal conservation planning has generally focused more on animals than plants, but, for example, the Conservation Breeding Specialist Group of IUCN has recently broadened the focus of its activities and changed its name to the Conservation Planning Specialist Group (http://www.cpsg.org/), and some publications now contain sections specifically focused on plants (e.g. IUCN 2017). The Orchid Specialist Group of IUCN looks forward to working with CPSG and similar organizations in developing effective conservation planning for orchids.

## Systematics and genetics: helping to set conservation priorities

If we are to conserve biodiversity effectively, we first need to know what exists, and there is a clear role for systematic and taxonomic studies in circumscribing species and in identifying high priorities for conservation. This is particularly the case in taxonomically complex groups. In *Dactylorhiza*, Pillon et al. (2006) found greater phylogenetic and genetic diversity in the Caucasus and the Mediterranean Basin than in Western Europe (generally regarded as the centre of diversity for this genus), and they stated that conservation of lineages of *Dactylorhiza* in the Caucasus and Mediterranean should be given greater priority; because species number is correlated with taxonomic effort, it is not always an appropriate measure of biodiversity as it can be sensitive to “taxonomic inflation” in well studied areas. They also stressed the importance of conserving areas where allotetraploids are formed rather than conserving specific allotetraploids, thus conserving process rather than named taxa (see also Ennos et al. 2012). In *Ophrys*, there is little consensus regarding the number of species that should be recognised, with authors accepting < 20 species (Pedersen and Faurholdt 2007) or > 300 species (Delforge 2006); setting conservation priorities with so little consensus is impossible, and further studies aimed at sorting out the relationships and delimiting the units for conservation are badly needed.

Many groups of orchids are still poorly known, especially in tropical regions, and phylogenetic studies will be necessary to identify the number of species to be recognized and those that are phylogenetically isolated and consequently of high conservation value. Borba et al. (2014), for example, showed that the rare and poorly known monospecific genus *Cotylolabium* from Brazil falls in an isolated position as sister to the remainder of subtribe Spiranthinae, and it should thus be treated as a high priority for conservation as it represents the same amount of phylogenetic history as the other c. 40 genera in the subtribe. On a wider scale, Li et al. (2018) investigated the use of phylogenetic measures as a means of prioritizing members of Orchidaceae for conservation in the Indo-Burma Biodiversity Hotspot, revealing Thailand, South China and Vietnam as the areas harbouring the highest phylogenetic diversity and *Tropidia curculigoides*, *Thaia saprophytica* and *Risleya atropurpurea* as accounting for disproportionately great evolutionary distinctiveness.

At the population level, genetic studies can be used to identify regions or populations that should be treated as high priority for conservation. This is a rapidly developing area and reviews become outdated quickly and will not be dealt with in detail here. Previously, development of markers was time-consuming and expensive, but new technologies are speeding up marker development and allowing more loci to be studied than previously possible, and the quality of the information to be used in conservation planning will improve as a result (e.g. Gargiulo et al. 2018).

## Conserving habitats

So long as climate change does not make conditions unsuitable for species, conserving the habitats where orchids and, for epiphytes, their host trees grow should be treated as the highest priority, and some countries have established reserves specifically for orchids (see, e.g., Cribb et al. 2003). Rasmussen and Rasmussen (2018) reviewed the relationship between epiphytic orchids and their host trees, calling for further research into the mechanisms controlling distribution of orchids on different species of trees.

Some orchid conservation organisations have the principle aim of establishing reserves, one being the Orchid Conservation Alliance (OCA), who state that “preservation of natural orchid habitat preserves the orchids, their pollinators, their genetic diversity, and other fauna, as well as the birds, frogs, insects, reptiles, and mammals in the forests where they live” (OCA 2017). However, this will not in itself be sufficient, given the pressures that orchids face from habitat destruction, unsustainable harvesting and climate change. Meeting these challenges will in many cases also involve a combination of creating new habitats, transplantation and ex situ conservation in seedbanks and living collections. Papers in this issue by Kendon et al. (2017), Zettler et al. (2017) and Higaki et al. (2017) focus on some aspects of ex situ conservation.

Conserving orchids in isolation from their pollinators, fungal associates and host plants means that the complexity of their biology is lost, even though the species still exists. For this reason, orchid conservationists are some of the keenest advocates of “integrated conservation”, using ex situ techniques to support in situ conservation as appropriate.

## Understanding pollinators and pollination

Orchids are renowned for the wide range of pollination mechanisms and syndromes (e.g. Darwin 1862; Micheneau et al. 2009) and the species diversity in the family has been attributed, in part, to the diversity of pollen mechanisms (e.g. Cozzolino and Widmer 2005). Because of the diversity of pollination mechanisms, Roberts (2003) stressed the importance of understanding pollination biology for effective orchid conservation, stating that “orchid conservation will require a case by case, functional ecosystem approach”, and noted the need to conserve not only the orchid and the pollinator, but also in some cases the “pollinator food source, nesting site, larval host species, and in the case of parasitic pollinators, the larval host plant of its host species”. If pollinators are not present in sufficient numbers (or at all), fruit production can be limited or absent, and this can have a major impact on the choice of sites for reintroduction programmes (Reiter et al. 2017). Hutchings et al. (2018) showed that climate change can decouple the phenology of pollinator and orchid species, potentially leading to reproductive failure of the orchid.

Despite the identified need for knowledge of the components necessary to ensure that pollinators are present and available for pollination, we are still far from understanding the pollination biology of many orchid species, and, despite the long history of the study of orchid pollination, new discoveries are still being made on a regular basis. Recent papers have investigated birds (e.g. Micheneau et al. 2006; van der Niet et al. 2015), crickets (Micheneau et al. 2010), fungus gnats (Phillips et al. 2014) and biting midges (Bogarín et al. 2018) as specialized pollinators. Many orchid species attract pollinators by deceipt, with the forms of deception including food deception, brood-site imitation, shelter imitation, rendezvous attraction and sexual deception (Jersáková et al. 2006), and recent discoveries of dual deceipt (pseudopollen lacking food value; Davies et al. 2013), carrion mimicry (van der Niet et al. 2011) and production of fruitfly aggregation pheromones (Karremans et al. 2015) demonstrate that we are far from understanding the full complexities of orchid pollination.

## Understanding mycorrhizal associations

Seed and protocorm development is reviewed in this issue by Yeung (2017), including discussion of mycorrhizal associations and the survival of orchid seeds and plantlets in their natural habitats. Clearly, the role of mycorrhizal fungi is crucial to the survival of self-sustaining populations of orchids, but there is much research still to be conducted before we fully understand the mycorrhizal associations, especially with epiphytic orchid species. Even with temperate terrestrial species, the processes involved are not fully understood, but techniques including measurement of isotope enrichment of carbon, nitrogen and hydrogen now allow us to demonstrate the contribution that the fungi make to the nutrition of orchids, even when the plants are apparently capable of photosynthesis (e.g. Gebauer et al. 2016). These techniques were recently used, for example, by Fay et al. (2018) to demonstrate that reintroduced seedlings of *Cypripedium calceolus* had established mycorrhizal associations after planting out, despite being produced axenically.

## Controlling trade: can harvesting of orchids from nature be sustainable?

With habitat destruction and global change, collection of orchids for horticulture, food or medicine represents one of the major threats to the survival of some groups of orchids, and Hinsley et al. (2018) highlighted four key priorities to address this problem. Much of the harvest of orchids and subsequent trade is unregulated and undocumented, and research into trade dynamics and the impacts of harvest will be of critical importance if we are to prevent orchids being driven into extinction. Strengthening the legal trade and tackling illegal trade were both identified as priorities—unless we provide the necessary support for the legal trade, the illegal trade will continue. Finally, raising the profile of orchid trade among policy makers, conservationists and the public was stressed by Hinsley et al. as being fundamental in underpinning the other priorities.

## Conclusions

Due to their complex life histories, orchids are liable to be severely affected by habitat destruction and climate change, and unsustainable (often illegal and undocumented) harvest presents a major additional risk to some groups of orchids. To conserve orchids effectively, we will need to understand their biology, and this will require further research into areas including pollination, mycorrhizal associations, population genetics and demographics. However, due to the large number of species involved, species-by-species approaches will only be feasible for those species identified as the highest priorities (due, e.g., to phylogenetic distinction, extreme rarity or narrow distribution) or at the greatest risk, and we will need to complement these with broader-scale approaches. These should include habitat conservation, especially for orchid-rich environments, conservation planning for groups of species (e.g. those that are closely related, affected by similar threats or growing sympatrically) and increased monitoring and control of harvest to ensure that this is legal and sustainable. Without such combined approaches, we will not be able to ensure the survival of these charismatic species into the future.
